# Genome-Wide mRNA Expression Correlates of Viral Control in CD4+ T-Cells from HIV-1-Infected Individuals

**DOI:** 10.1371/journal.ppat.1000781

**Published:** 2010-02-26

**Authors:** Margalida Rotger, Kristen K. Dang, Jacques Fellay, Erin L. Heinzen, Sheng Feng, Patrick Descombes, Kevin V. Shianna, Dongliang Ge, Huldrych F. Günthard, David B. Goldstein, Amalio Telenti, The Swiss HIV Cohort Study and the Center for HIV/AIDS Vaccine Immunology

**Affiliations:** 1 Institute of Microbiology, University Hospital and University of Lausanne, Lausanne, Switzerland; 2 Institute for Genome Sciences & Policy, Duke University, Durham, North Carolina, United States of America; 3 Department of Biostatistics and Bioinformatics, Duke University, Durham, North Carolina, United States of America; 4 Genomics Platform, University of Geneva, Geneva, Switzerland; 5 Division of Infectious Diseases, University Hospital Zurich, University of Zurich, Zurich, Switzerland; Fred Hutchinson Cancer Research Center, United States of America

## Abstract

There is great interindividual variability in HIV-1 viral setpoint after seroconversion, some of which is known to be due to genetic differences among infected individuals. Here, our focus is on determining, genome-wide, the contribution of variable gene expression to viral control, and to relate it to genomic DNA polymorphism. RNA was extracted from purified CD4+ T-cells from 137 HIV-1 seroconverters, 16 elite controllers, and 3 healthy blood donors. Expression levels of more than 48,000 mRNA transcripts were assessed by the Human-6 v3 Expression BeadChips (Illumina). Genome-wide SNP data was generated from genomic DNA using the HumanHap550 Genotyping BeadChip (Illumina). We observed two distinct profiles with 260 genes differentially expressed depending on HIV-1 viral load. There was significant upregulation of expression of interferon stimulated genes with increasing viral load, including genes of the intrinsic antiretroviral defense. Upon successful antiretroviral treatment, the transcriptome profile of previously viremic individuals reverted to a pattern comparable to that of elite controllers and of uninfected individuals. Genome-wide evaluation of *cis*-acting SNPs identified genetic variants modulating expression of 190 genes. Those were compared to the genes whose expression was found associated with viral load: expression of one interferon stimulated gene, *OAS1*, was found to be regulated by a SNP (rs3177979, p = 4.9E-12); however, we could not detect an independent association of the SNP with viral setpoint. Thus, this study represents an attempt to integrate genome-wide SNP signals with genome-wide expression profiles in the search for biological correlates of HIV-1 control. It underscores the paradox of the association between increasing levels of viral load and greater expression of antiviral defense pathways. It also shows that elite controllers do not have a fully distinctive mRNA expression pattern in CD4+ T cells. Overall, changes in global RNA expression reflect responses to viral replication rather than a mechanism that might explain viral control.

## Author Summary

There has been recent progress in understanding the genetic factors that modulate susceptibility to HIV-1 infection. Genetic variation explains to a certain extent differences in disease progression among individuals. Less is known regarding the contribution of differences in gene expression to viral control. The present study evaluated, genome-wide, gene expression levels in CD4+ T cell, the main target of HIV-1. Thereafter, it searched for genetic variants that would modify gene expression. Specific expression profiles associated with high levels of viremia—in particular, the upregulation of genes of the antiviral defense. In contrast, no expression profile associated with effective viral control. Multiple genetic variants modulated gene expression in CD4+ T cells; however, none had a strong influence on viral control. This integrated genome-wide assessment suggests that viral replication drives gene expression rather than expression pointing to mechanisms of viral control.

## Introduction

There has been a recent effort to identify the genomic determinants of susceptibility to HIV-1 infection, control of viral replication, and disease progression [1]. Genetic analyses have identified over the years a number of validated variants in candidate genes, while a recent genome-wide association study [2] highlighted the dominant role of variants in the MHC region in the control of viral setpoint (the steady state of viral replication after infection) and disease progression. Other genome-wide studies [3]–[5] confirmed the variants identified in the first genome-wide analysis. These variants collectively explain up to about 13% of the variation in viral setpoint, indicating that other biological determinants of control have yet to be identified. Here our focus is on determining the contribution of variable gene expression to viral control, and to relate it to genomic DNA polymorphism.

There have been a number of transcriptome studies in HIV-1 target cells (CD4+ T cells, monocytes/macrophages), non-targets such as NK cells and B cells, and of dendritic cells and total peripheral blood mononuclear cells (PBMCs) (reviewed in [6], and recent publications [7]–[11]). These studies provide insight into gene expression changes associated with virus replication and persistence. Studies are limited by the number of genes interrogated, or by the number of individuals investigated. These limits notwithstanding, microarray data have yielded novel mechanisms of HIV-mediated pathogenesis. Transcriptome analyses of cell lines transfected with individual viral proteins or mutant viruses have also been reported (reviewed in [6]).

This study aims at coupling a large scale assessment of gene expression in purified CD4+ T cells from HIV-1 infected individuals, with genome-wide genotype data tested for association with viral setpoint. Integrating gene expression data with results from genome-wide association studies may help prioritize fine-mapping efforts and provide shortcuts to disease biology [12]. Therefore, the goals of the study are the description of the expression program associated with HIV-1 *in vivo*, the identification of mRNAs that are differentially expressed in individuals that present effective control of viral replication, and the search for *cis*-acting variation in differentially expressed genes. Expression polymorphism due to single nucleotide polymorphisms (SNPs) that influence mRNA levels has received increasing attention for the understanding of phenotypes in health and disease (reviewed in [12]). Genome-wide screens, most generally done in cell lines, have established the relevance of *cis*-acting SNPs in expression polymorphism [13]. However, little is known regarding expression polymorphism and HIV-1 disease.

In order to have power to detect correlations, we have considered a large sample set of purified CD4+ T cells from individuals with known date of infection and carefully determined viral load results. Transcription analysis was done at the time of viral setpoint, so that samples are representative of the steady-state replication for a given individual, and across the full range of viral setpoint in an infected population. For a large subset of participants, we also established the transcription profile after initiation of antiretroviral therapy (ART) to assess the modulation of expression upon effective control of viremia. Thus, this study represents a first attempt at assessing, genome-wide, the genotype-to-transcriptome-to-clinical phenotype associations in HIV-1 disease.

## Results

### Transcriptome profile and viral setpoint analysis

**Figure 1:**
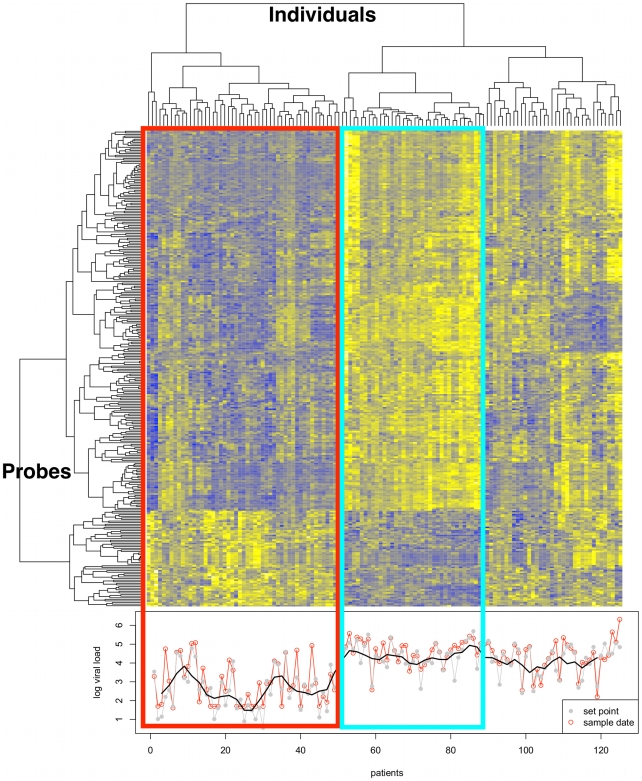
Transcriptome analysis in CD4+ T cells from HIV-infected untreated individuals. Gene clusters are presented on the left. In total, 260 genes are differentially expressed (at adjusted p<0.01) in association with viral load in CD4+ T cells during *in vivo* HIV-1 infection. Patient clusters are presented at the top for untreated individuals. Clustering was performed on the Spearman correlation coefficient. The phenotype is presented at the bottom, as log10 viral setpoint in gray, and log10 viral load at time of sample collection in red. A smooth of the setpoint viral load values is depicted by the black line. The red rectangle surrounds a cluster of individuals characterized by low viral load (mean Log10 viral setpoint = 2.6), and including several “elite controllers” – individuals that spontaneous control viral replication in the absence of treatment. The blue rectangle identifies a cluster of individuals with high viral setpoint (mean Log10 viral setpoint = 4.4). The remaining clusters illustrate the heterogeneity of transcription profile across the range of viral load values.

We identified 298 hybridization probes that were significantly correlated with viral load (FDR-adjusted p-value<0.01). This resulted in a list of 260 genes, since multiple probes are used for some of the genes. The majority of these (n = 209) were positively associated with viral setpoint, while a smaller group (n = 51) was negatively associated (**Supplementary Table S1**). We used (unsupervised) clustering to group the expression profiles of the samples for these 260 genes, and found that they showed distinct behavior in individuals with effective virus control (reflected in low viral setpoint) as compared with individuals showing poor control of viral replication (**Figure 1**). In an analysis that considered viral load at the precise date of transcriptome analysis instead of setpoint, the results were comparable, implying that the expression profile is representative for the period of analysis (three months to three years after seroconversion), **Figure 1**. The analysis included various parameters as covariates (clinical center, gender, age, CD4 T cell viability and laboratory date, and microarray chip batch - sentrix ID). The CD4 T cell value at the time of sampling was found to be closely correlated with viral setpoint (Pearson's correlation of −0.5 and a p-value = 1.195e-10), which made difficult to separate their effects on the data. The 149 genes that are shared between analysis using CD4 T cell count, and the analysis using viral setpoint are indicated in **Supplementary Table S1**.

**Figure 2:**
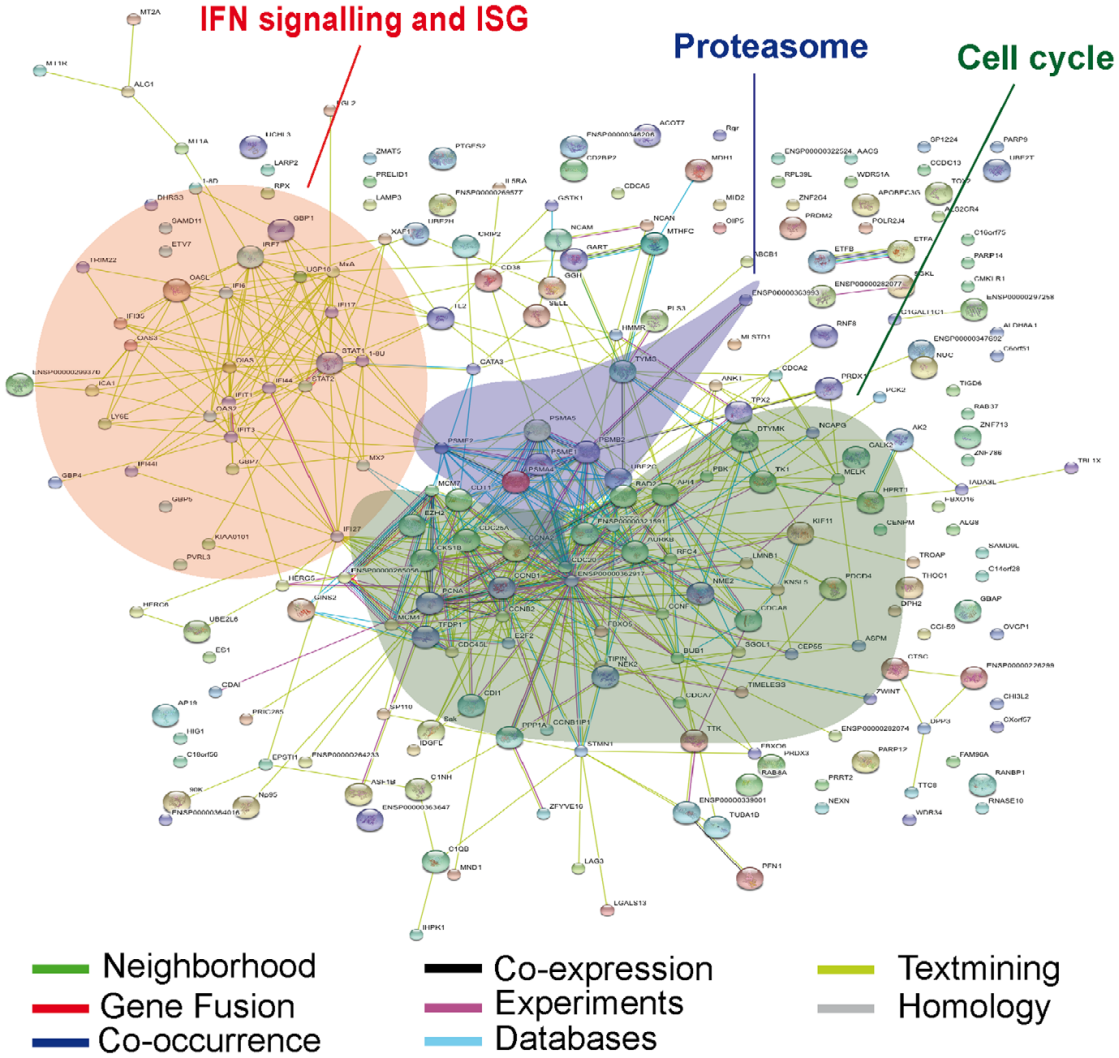
Predicted interaction networks of genes differentially expressed during HIV-1 infection. Differentially expressed genes are depicted: links have been predicted using STRING (http://string.embl.de/). Predicted interactions are depicted according to the type of available evidence. The interactions (see color labels) include direct (physical) and indirect (functional) associations; they are derived from four sources: genomic context, high-throughput experiments, conserved coexpression, and previous knowledge from literature.

The main gene clusters exhibiting a positive correlation with viral setpoint (i.e., increasing gene expression with increasing viral load), as defined by STRING, DAVID and IPA, were the interferon pathway, the proteasome, and cell cycle genes (**Figure 2** **and Supplementary Table S2, S3**). Conversely, among genes that exhibited a negative correlation with viral setpoint (**Supplementary Table S1B**), no pathway enrichment was identified. A separate analysis that used a gene-by-gene modeling approach resulted in a list of significant genes that was shorter (44 genes) but highly concordant with the output of the empirical Bayes analysis described above (**Supplementary Table S4**): we therefore used the empirical Bayes results for subsequent analyses. Because the CD4+T cell composition may vary depending on the degree of viral replication [7], we re-analyzed the data controlling for *CD25* expression (encoding IL2RA as marker of activation), or *CD62L*, *CD40L*, *CD11a*, and *CD27* (markers that distinguish naive from memory CD4+ T cells). Although several additional significant genes were found using each of the above markers as covariates, the overall expression profile did not vary significantly (see for example data from analysis adjusted by *CD25* in **Supplementary Table S5**). These analyses indicate the existence of a clear expression program associated with high viral load, but fail to identify definite gene networks associated with viral control.

### Analysis of genes of the interferon response pathways

**Figure 3:**
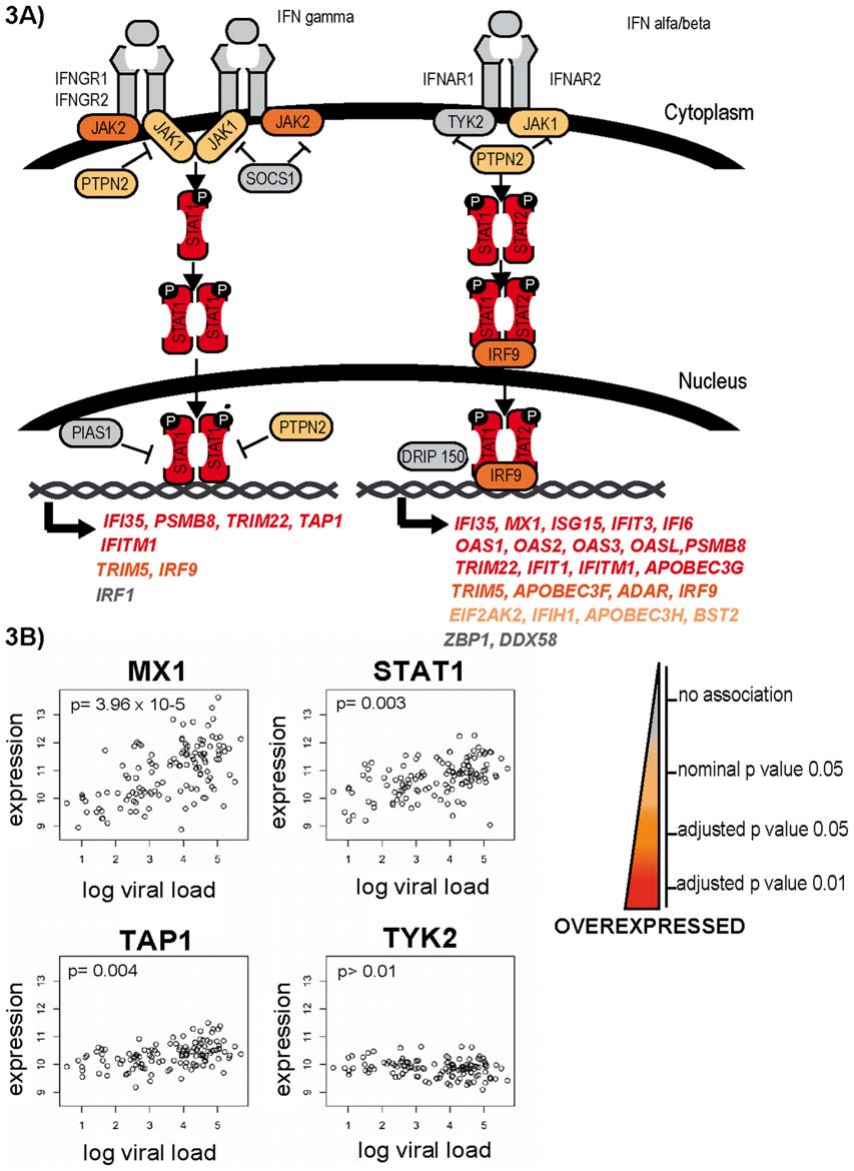
Differential expression of genes of the interferon response. Representative genes of the interferon response pathway are shown in panel A. From grey to red, increasing differential expression with increasing viral setpoint. Selected genes are shown in panel B. While genes associated with interferon receptors, such as *TYK2*, are not differentially expressed, signaling molecules such *STAT1* and interferon-stimulated genes such as *MX1* and *TAP1* are significantly upregulated with increasing viral load.

We observed a linear association between increasing expression of interferon signaling and interferon-stimulated genes (ISGs) and increasing viral setpoint. We compiled a list of 40 genes implicated in the interferon response [14] (**Supplementary Table S6**). Seventeen genes were significantly associated with viral setpoint after FDR adjustment at the 0.01 level, and 12 were associated at a p-value of 0.05. These 29 genes comprise most of the signaling and ISGs, but notably exclude the interferon genes themselves and the interferon receptors (**Figure 3**). This analysis points to a de-regulated interferon response that associates with an ineffective antiviral response.

### Analysis of genes associated with HIV-1 life cycle and pathogenesis

We similarly examined in detail a list of selected genes reported to be involved in HIV-1 life cycle or pathogenesis (see Methods for explanation of candidate selection) [15]. Of this list, 138 genes were matched to probes, with four having a FDR-adjusted significant association with viral setpoint, p-value <0.01: *TRIM22*, *IRF7*, *RANBP1*, and *APOBEC3G*. An additional 12 genes had FDR-adjusted p-values <0.05, and a further 26 had nominal p-values <0.05 (**Supplementary Table S7**). Genes of the intrinsic cellular defense against retroviruses (*TRIM5α*, *TRIM22*, *TRIM19/PML*, *APOBEC3G*, *APOBEC3F*, *APOBEC3H*, *PPIA/Cyclophilin A*, *BST2/Tetherin*) were all upregulated with increasing viral load, which is consistent with their general dependence on the interferon pathways. A number of chemokines and chemokine receptors were also positively modulated with increasing viremia. We also identified differentially expressed genes that are present in both the current analysis and studies that used siRNA or shRNA to identify HIV-1 dependency factors [16]–[19] (**Supplementary Table S8**).

### Changes in transcriptome profile after treatment

The significant association of a number of genes and pathways with viral setpoint was further assessed by observing the changes in transcriptional profile in CD4+ T cells after viral suppression. We found statistical support for differential expression of 247 probes (FDR-adjusted p-value <0.01) between treated and untreated-noncontroller individuals. The list of genes involved had an extensive overlap with the list of genes associated with viral setpoint in the transcriptome analysis above (**Supplementary Table S9**). The list also shares 97 genes with the recent study by Li et al. [20] on changes in the lymph node transcriptome profile upon initiation of ART. This analysis indicates that successful treatment appears able to recapitulate the cellular state of a well-controlled individual, since we did not find support for any probes being differently expressed between successfully treated and untreated-controller individuals.

### Comparison with uninfected individuals

**Figure 4:**
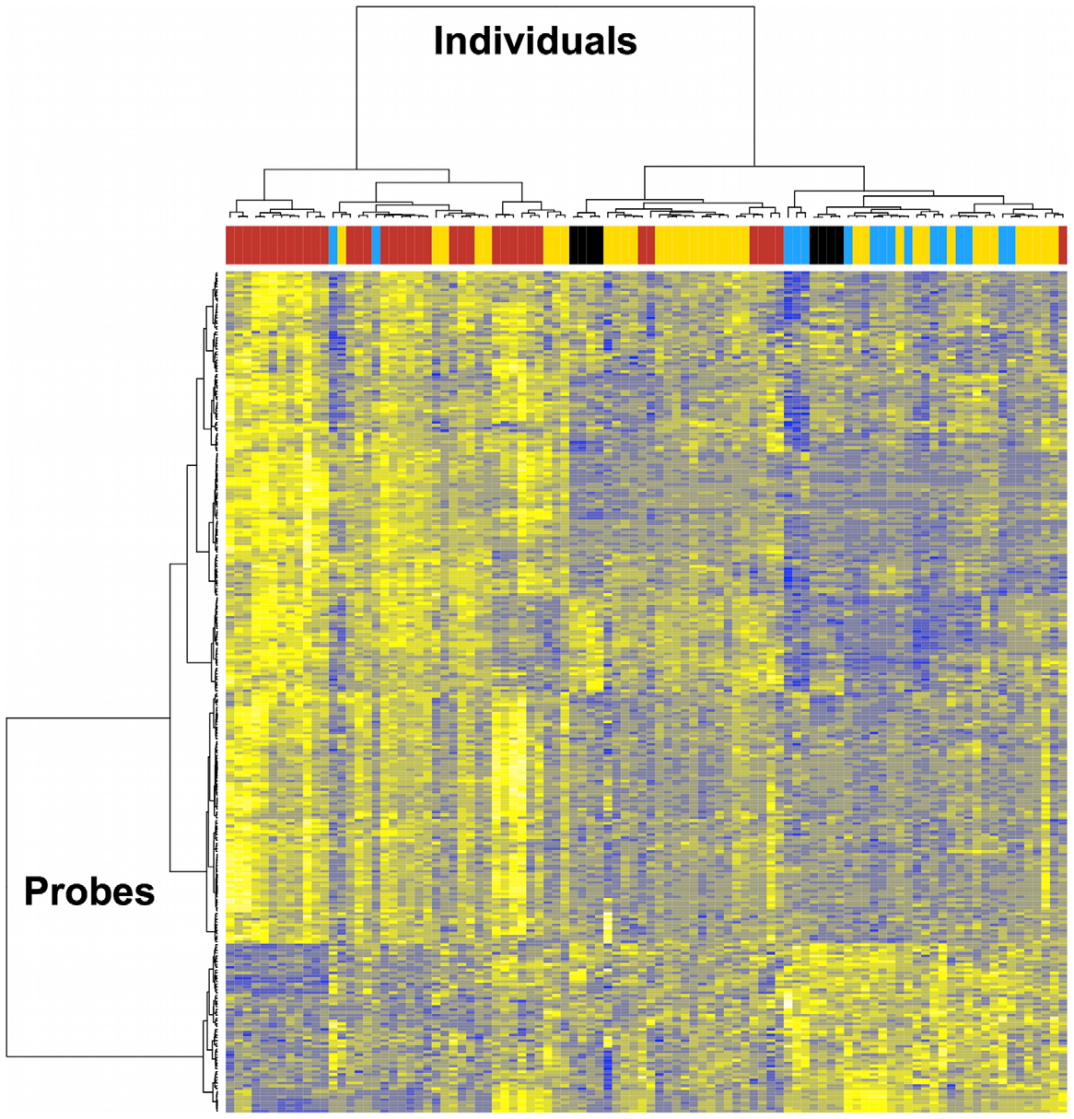
Transcriptome analysis in CD4+ T cells from HIV-infected individuals before and after viral suppression. Analysis was restricted to the 260 genes found to be differently expressed by viral setpoint. Gene clusters are presented on the left. Patient clusters are presented at the top. In red, transcriptome profile before viral suppression, and in yellow, transcriptome profile after viral suppression with effective treatment in 37 individuals with pre- and post-treatment initiation samples. In blue, transcriptome profile of 16 elite controllers. In black, transcriptome profile from 3 HIV-negative healthy controls (8 samples).

To compare the treated and untreated individuals with uninfected individuals, we clustered the expression profiles from samples from a selected group of individuals, including elite controllers (viral load <50 copies/ml), samples from successfully treated individuals and their paired untreated samples, and from the three uninfected individuals (measured in triplicate, one triplicate failed analysis). For this, we restricted analysis to the 260 genes found to be differently expressed by viral setpoint. As shown in **Figure 4**, both the successfully treated and uninfected individuals tended to cluster with the controllers individuals. Two of the individuals from healthy donors were most tightly grouped with several of the untreated individuals that have the lowest level of virus at setpoint (i.e. the elite controllers), while one uninfected individual showed a profile that is less extreme, but still most similar to the viral control profile. Treated individuals also preferentially grouped with the viral control pattern, although the majority showed a mid-range expression level and a smaller fraction grouped with elite controllers and uninfected individuals. A bootstrapping analysis showed support (p-value 0.06) for the consistency of the top-level groupings with one group containing all the uninfected individuals and the majority of the treated and elite controller individuals, while the other group contained mostly non-controller individuals. This indicates that the expression levels of individuals with the best viral control closely resemble those of uninfected individuals.

### Screen for *cis*-acting SNPs regulating transcript expression levels

Among a total of 1.3 million association tests comparing 399,626 gene-centric SNPs (some SNPs were within 100 kb of multiple transcripts) with 28,828 individual probes measuring a total of 18,059 unique transcripts, we detected 782 study-wide significant associations (SNP-probe associations) below the threshold p-value of 3.8×10−8. Stepwise linear regression was used to prune out redundant associations of SNPs with a particular probe. This step resulted in evidence for *cis*-regulation of 208 unique probes, 157 of which were regulated by multiple SNPs in high linkage disequilibrium between SNPs included in the analysis (51 signals of unique SNPs with a transcript, and 731 signals arising from the regulation of 157 transcripts by multiple non-unique SNPs). These 208 associations included 193 SNPs that modulate 190 genes in CD4+ T cells, with the overlap occurring because of probe cross-hybridization, and also several probes detecting the same gene (**Supplementary Table S10**).

This list of study-wide significant associations was compared to the list of genes whose expression was found associated with viral load at setpoint. Among genes under differential expression during HIV-1 infection, several showed evidence for *cis*-regulation (**Supplementary Table S11**) but only one, involving the interferon stimulated *OAS1*, reached study-wide significance. *OAS1* was found to be regulated by an intronic SNP (rs3177979) located near exon 6 (**Supplementary Figure S1**). Lower expression was associated with the rs3177979 GG genotype. The association was detectable in treated and untreated individuals; however the expression level was lower in samples from treated individuals. The association of this SNP with *OAS1* transcript expression is also detectable in PBMCs collected from uninfected controls [21].

We did not observe an association of *OAS1* rs3177979 with viral setpoint in the study (untreated) population. However, given the potential interest of genetic polymorphism in *OAS1*, we also assessed the association between rs3177979 and HIV-1 outcomes in a large population of 2362 individuals [5]. The association p-values were 0.05 for an association of the *OAS1* SNP and viral setpoint and 0.09 for HIV-1 disease progression, but differences were subtle: mean HIV-1 load was 4.11 log10 viral copies/ml for the AA genotype, 4.07 for AG, and 4.01 for GG. Because rs3177979 is in linkage disequilibrium with rs10774671, a SNP associated with a splicing variant ([22] and **Text S1**) reported to have greater activity against West Nile virus [23], we re-genotyped the population for this putative functional SNP, without finding any stronger association: we have therefore no definitive evidence of an association of *cis*-acting genetic variation in *OAS1* with HIV-1 viral control or disease progression.

One additional gene, *RANBP1*, encoding a Ran GTPase-binding protein that interferes with Rev-mediated expression of HIV-1 [24], presented both increased expression at higher setpoint, and a *cis*-acting SNP (rs2008591) that modulates its expression (**Supplementary Table S11**). We assessed the association between rs2008591 and viral setpoint and disease progression in the large population of 2362 individuals [5]. Here, rs2008591 did not associate with viral setpoint (p = 0.45) or disease progression (p = 0.35). Overall, these analyses identified a significant number of *cis*-acting genetic variants influencing gene expression in CD4+ T cells; however, expression polymorphism, genome-wide or among genes that are modulated during HIV-1 infection, did not contribute in a significant fashion to viral control.

## Discussion

This study represents the largest effort to date to characterize the mRNA expression profile in CD4+ T cells *in vivo* in HIV-1 infected individuals. The study population, only including individuals with known date of seroconversion or elite controllers, represents the complete range of viral load control: from undetectable viral load to sustained high levels of viral replication. The study also analyzed changes in transcriptome upon successful antiretroviral therapy. In addition, we searched for *cis*-acting variants – SNPs that would possibly associate with the observed differences in gene expression in the course of HIV-1 infection. Overall, changes in RNA expression reflect responses to viral replication rather than a mechanism that might explain control of viral replication. As such, the reactive transcriptome profile we observed shares common responses with other viral infections, eg. to dengue virus [25]–[28] (**Supplementary Table S12**).

*In vivo* HIV-1 infection results in a distinctive mRNA transcriptome profile in CD4+ T cells that involves 260 genes in an analysis that differentiates individuals with high and those with low viral setpoint. Under conditions of high viral load, there is a distinct upregulation of the interferon pathways, cell cycle and the ubiquitin-proteasome degradation machinery. The study confirms and extends previous analyses of *in vitro* infection of T cell lines, or of CD4+ T cells *in vivo* that were performed on a limited number of individuals [7]–[10],[29],[30].

This study underscores that the observed increase in transcription of ISGs is not associated with a better control of viremia [7]. This contrasts with the reported efficacy and possible therapeutic role of interferon (IFN-α, IFN-α2β) suggested by results from *in vitro* studies, while exogenous administration of interferon in clinical trials led to doubts about its efficacy in the clinical setting (reviewed in [31]). Our observations lend support to the hypothesis that interferon activation plays a deleterious role in retroviral pathogenesis, as proposed by many recent reports (reviewed in [31]). Elevated ISG expression is associated with disease progression in pathogenic SIV infection of non-human primates [32]–[35], while the type I interferon response subsided after peak viral load during non-pathogenic infection [36],[37]. Sedaghat et al. [7] compared the transcriptional programs of *in vivo*-activated CD4+ T cells from untreated HIV-positive individuals with those of activated CD4+ T cells from HIV-negative individuals. From this study, they concluded that CD4+ T cells from infected individuals are in a hyperproliferative state that is modulated by type I interferons, and that this would lead, during chronic infection, to CD4+ T-cell preferential differentiation and depletion. Imbeault et al. [10] suggested that interferon could lead to a sustained increase in p53 mRNA levels and therefore to a higher susceptibility of CD4+ T cells to pro-apoptotic signals. Herbeuval and Shearer [31] proposed that interferon, through binding to its receptor on primary CD4+ T cells would result in membrane expression of the TNF-Related Apoptosis-Inducing Ligand, TRAIL, death molecule leading to the selective death of HIV-exposed CD4+ T cells. More recently, Sato et al. [38] showed that type I interferon induce proliferation and exhaustion in hematopoietic stem cells; chronic and excessive type I interferon signaling may cause hematopoietic stem cells reduction. Overall, interferon response appears a poorly effective antiretroviral mechanism, and may actually contribute to HIV-1 disease [7],[39].

Among genes previously associated with HIV-1 pathogenesis, the analysis identified a number of significant associations, in particular for genes of the intrinsic cellular defense against retroviruses. Many of these respond to interferon, and thus have the same profile of increased expression with increasing viral load as ISG. Thus, these genes appear ineffective both by their poor specificity against HIV-1 and by the apparent limited response of HIV-1 to increasing titration of the transcripts. We also analysed genes issued from four genome-wide siRNA/shRNA screens [16]–[19]. Fifteen genes that were associated with decreased cellular permissiveness to infection after silencing, were upregulated with increasing viremia *in vivo* in the current study. They deserve further inspection for a role in HIV-1 pathogenesis. Although the scope of the present work was not to complete a meta-analytical study of all available genome-wide transcriptome studies and siRNA screens [40], we are aware of the interest to progressively integrate large scale datasets [41].

We aimed at identifying patterns of gene expression associated with effective viral control. However, the nature of the analysis could not establish whether high levels of viral replication would lead to the observed transcriptional profile, or whether genetic modifiers of transcriptional profile were determinants for the control of viral replication. This was addressed first by comparing the transcriptional profile of CD4+ T cell from elite controllers with that of successfully treated individuals and healthy donors. Here, we observed that the expression profile of genes associated with active viral replication was, after effective treatment, similar to that of individuals with spontaneous control of viral replication, and close to that of healthy donors. This suggests that infection drives gene expression rather than the contrary. Second, we tested the hypothesis that genetic variants influence expression levels of genes, thus leading to differences in viral control. The analysis identified a number of variants that would possibly act in *cis* to modulate gene expression – most notably a variant in *OAS1* that has been associated with improved control of West Nile virus infection [23]. It may be argued that if a variant influences expression of a gene, and if expression of that gene correlates with viral load, then the two analyses will be partially redundant. However, we emphasize that this approach allows for independent information because the variation in expression of few if any genes is determined exclusively by *cis*-acting variants. In addition, the identification of strong *cis*-acting variants would contribute to disentangle causation and correlation. Thus if a gene expression correlates with viral load, it could be that the change in expression is a response to the amount of virus, or it that the gene directly controls the viral level. In the former case, a *cis*-acting variant will show no association with viral load, whereas in the latter it will. In the present study, none of the candidate *cis*-acting SNPs, or SNPs in the implicated genes was associated with differences in viral setpoint in a genome-wide association analysis. These results do not contradict current evidence of mechanisms of viral control through differences in expression levels of particular genes, most notably CCR5 [42]. Rather, the analysis indicates that polymorphisms in genes implicated in the differential expression programs do not represent a strong source of variation at the population level.

There are a number of technical and conceptual limits to the study. The study failed to identify a transcriptome profile characteristic of elite controllers. This may be attributed to the large scale approach, as the current technology covers a total of 25,440 annotated human genes. While this allows for pathway or network analyses, it may fail in the identification of subtle expression changes, in particular at the level of the individual gene. On one hand, the analysis would require greater study power (ie, additional elite controllers) to compensate the penalty of correction for multiple testing. On the other hand, the precision of several of analyses described earlier in this section could be improved through the added resolution of new technology such as RNA-Seq [31], or the targeted multiplexed measurement of gene expression in selected pathways [43]. High-throughput deep sequencing results in a superior dynamic range, and allows quantitative analysis of coding and non-coding region transcripts, such as small RNAs. It should also be pointed out that the use of cryopreserved cells may result in changes in the transcriptome and in transcript stability. However, this allowed the investigation of a large number of samples from seroconverting individuals in batch analyses. We argue that the internal consistency of the results and the general agreement across studies supports the robust nature of the transcription profiles that were generated.

In conclusion, while this study suggests that the generalized upregulation of ISG, an important component of viral defense, does not lead to consistently improved viral control throughout the course of infection, it does not implicate any specific gene expression network in viral control. There are several possible explanations for these observations. First, the most important cellular populations for determining control may be effector cells such as CD8+ T cells or NK cells whose expression patterns have not been evaluated here. Second, the key expression patterns that determine eventual control may be only detectable early in infection and thus largely missed in studies focusing on cells taken during the setpoint period. These possibilities argue strongly that the next phase of expression work in the study of HIV-1 control must focus on large scale analysis of isolated populations of effector cells taken from individuals as early in the course of infection as possible and in a standardized fashion. We believe the approach taken here provides a general template for such studies.

## Materials and Methods

### Ethics statement

Study participants were followed in the Swiss HIV Cohort Study (www.shcs.ch). The Genetics Project of the Swiss HIV Cohort Study was approved by the ethics committees of all participating centers, and the permission for genomic work was approved by the Institutional Review Board/Ethics Committee of the University Hospital of Lausanne. Participants gave written, informed consent for genetic testing.

### Participants

198 HIV-1 infected individuals from the Swiss HIV Cohort study with a known date of seroconversion (n = 182), or elite controllers (n = 16) were included in the study. Seroconversion was defined on the basis of a documented positive test and date and a documented negative test less than two years before the first positive test. The viral setpoint was calculated for each participant by using a median of 4 (range 2 to 8) plasma HIV-1 RNA determinations obtained in the absence of antiretroviral treatment between 3 months and 3 years after seroconversion, as previously described [2]. See **Text S1** for the detail definition of viral setpoint and of elite controllers. When available, HIV-1 infected participants contributed samples during stable viral setpoint before and under effective ART (median [IQR] from treatment initiation to sample collection was 1297 (434–2730) days). In addition three healthy blood donors provided three control samples used as biological replicas. Quality control steps at the level of cellular viability, RNA integrity, microarray and hybridization quality, and data analysis led to a final number of 190 samples from 153 participants and 8 samples from 3 healthy controls (68% of valid samples, 78% of successful recruitment). The demographic characteristics of the patients and the flow chart of enrollment and sample validation is presented in **Text S1**. Representative examples of QC checks are presented in **Supplementary Figure S2**.

### Cell isolation and RNA extraction

CD4+ T cells were positively selected from frozen PBMCs (median time [IQR] of cryopreservation was 616 [333–1448] days) using magnetically labeled CD4 microbeads and subsequent column purification according to the manufacturer's protocol (Miltenyi Biotec). CD4+ T cell purity, verified by flow cytometry, was 95.6% (86.4–98.1%) [median (range)]. CD4+ T cell viability was assessed by the trypan blue dye exclusion method using the Vi-CELL (Beckman Coulter). Total RNA was extracted from purified CD4+ T cells using mirVana miRNA isolation kit (Ambion) according to the manufacturer's protocol for total RNA extraction. RNA amount was estimated by spectrophotometry using the Nanodrop 1000 (Thermo Fisher). RNA quality was determined by Agilent RNA 6000 pico kit on an Agilent 2100 Bioanalyzer. We used cryopreserved samples because of the interest to analyse a large population of seroconverting individuals during the precise window of stable viral setpoint. Samples were collected between 1995 and 2007, and investigated in 2008. The median (range) of CD4+ T cell viability for samples that were successfully analysed was 78.5% (IQR 70.5–85.3). Viability was minimally dependent on time of cryopreservation, and more dependent on collection center. These covariates were included in the analyses (see below).

### Transcriptome analysis and genome-wide genotyping

200 ng of total RNA was amplified and labeled using the Illumina TotalPrep RNA Amplification kit (Ambion). cRNA quality was assessed by capillary electrophoresis on Agilent 2100 Bioanalyzer. Expression levels of over 48,000 mRNA transcripts were assessed by the Human-6 v3 Expression BeadChips (Illumina). Hybridization was carried out according to the manufacturer's instructions. Genome-wide SNP data had been generated from genomic DNA using the HumanHap550 Genotyping BeadChip (Illumina) with 555,352 SNPs [2].

### Selection of candidate genes for subanalysis

We screened the literature for genes associated with biology of HIV-1 (reviewed in [44]–[47] and recent studies [15],[48],[49]), as well as HIV-1 dependency factors emerging from genome-wide siRNA screens [16]–[19], and genes considered polymorphic and involved in HIV-1 pathogenesis (compiled in www.hiv-pharmacogenomics.org). For the three large siRNA screens, that resulted in over 600 candidates, we restricted analysis to (i) genes identified in at least two of three screens, or to (ii) genes with SNPs that reached a nominal significant p value in a recent genome-wide association study of determinants of susceptibility to HIV-1 [2].

### Data pre-processing

Bead summary data was output from Illumina's BeadStudio software without background correction, as this has previously been shown to have detrimental effects [50]. Data pre-processing, including a variance-stabilizing transformation [51] and robust-spline normalization were applied as implemented in the lumi package [52] of R. Four outlier samples identified based on aberrant expression of control probes and aberrant median-interquartile range values compared to other samples were removed.

### Differential expression analysis

We applied an empirical Bayes analysis approach within a linear mixed-model framework to identify associations between variation in gene expression and in viral setpoint. The Empirical Bayes approach has been developed to model the variation profiles of all genes and use that information as prior knowledge to better estimate the variance of each gene expression [53]–[55]. In addition, we used a more conservative gene-by-gene modeling approach for result comparison with the empirical Bayes approach. We controlled for variation caused by gender, age, CD4+ T cell viability, location of sample collection, and laboratory batch effects. Effect of chip batch was modeled as a random effect; all others were fixed or continuous. All samples from untreated individuals were tested for association of expression with viral setpoint. We used a false discovery rate (FDR) method [56] to control for multiple testing. Probes selected for further analysis had an FDR-adjusted p-value <0.01. A separate analysis compared expression in samples from treated and untreated individuals, using a similar mixed-model approach as above, but also incorporating viral load as a factor in the analysis.

We tested for effect of treatment by separately comparing samples from treated individuals to each of the untreated groups, using the limma (linear models for microarray data) package in R with FDR adjustment as above. This analysis explicitly excluded samples from the same individuals because the statistical approach did not allow control for both the correlation between paired samples and the strong correlation (batch) effect of chip. To compare samples from treated and untreated individuals with samples from uninfected controls, we clustered the expression profiles for a selected group of individuals. We performed 1000 replicate clusterings on the Pearson correlation coefficient, using the “ward” clustering method as implemented in the pvclust package in R.

### Pathway and network analyses

The Search Tool for the Retrieval of Interacting Genes/Proteins (STRING) (http://string.embl.de/) was used to identify known and predicted interactions (derived from four sources: genomic context, high-throughput experiments, co-expression, and previous knowledge). DAVID Bioinformatic resources (http://david.abcc.ncifcrf.gov/) using the annotation sources GOTERM-BP (biological process), and GOTERM-MF (molecular function) identified functional categories [57]. Ingenuity Pathway Analysis 7.0 (IPA) (http://www.ingenuity.com/) was used for the analysis of pathway enrichment. Analysis was limited to genes significantly associated with viral load (FDR p-value <0.01).

### Screen for *cis*-acting SNPs regulating transcript expression levels in HIV-infected CD4+ T cells

Normalized expression data was exported for all untreated, HIV-1 infected individuals (n = 125). Only probes that targeted fully annotated genes were included in the analysis. A principal components analysis was run to assess batch effects. The *cis*-screen consisted of a scan for common SNPs, within 100 kb of the defined gene start and stop positions, for effects on transcript expression levels. The analysis was limited to SNPs with a minor allele frequency greater than 0.04, requiring at least ten alleles to be present to detect associations with a low false positive rate. This analysis was performed using a standard linear regression, incorporating age, gender, and 11 eigenstrat axes to correct for population stratification. In total, there were 1,330,529 tests run, therefore using a Bonferroni correction, a p<3.8×10^−8^ was used to declare a statistically significant association.

### Microarray data accession number

All microarray results have been deposited in the Gene Expression Omnibus database (GSE18233).

## Supporting Information

Figure S1 *OAS1* was found to be regulated by an intronic SNP (rs3177979) located near exon 6.
(7.16 MB TIF)

Figure S2 Quality control. Outlier samples were identified based on aberrant expression of control probes and aberrant median-interquartile range values.
(0.34 MB TIF)

Table S1 Genes differentially expressed (at adjusted p≤0.01) according to the empirical Bayes approach. (A) 209 genes that are upregulated at high viral load. (B) 51 genes that are downregulated at high viral load.
(0.06 MB XLS)

Table S2 Enrichment of biological process and molecular function determined using DAVID. (A) Enrichment for the 209 genes that are upregulated at high viral load. (B) Enrichment for the 51 genes that are downregulated at high viral load.
(0.04 MB XLS)

Table S3 Pathway enrichment for the 260 differentially expressed genes according to Ingenuity.
(0.02 MB XLS)

Table S4 Genes differentially expressed (at adjusted p≤0.01) according to the more conservative gene-by-gene modeling approach.
(0.02 MB XLS)

Table S5 Genes differentially expressed when analysis is adjusted by expression of *CD25* as covariate.
(0.08 MB XLS)

Table S6 List of interferon regulated genes.
(0.03 MB XLS)

Table S7 Analysis of expression profile of genes associated with HIV life cycle and pathogenesis.
(0.04 MB XLS)

Table S8 Genes identified in the current study that have been previously reported in genome-wide siRNA screens.
(0.02 MB XLS)

Table S9 Overlapping genes associated with difference in viral setpoint and associated with changes in transcriptome profile after treatment.
(0.04 MB XLS)

Table S10 Study-wide significant SNP-probe associations.
(0.32 MB XLS)

Table S11 Genes showing *cis*-regulation (eQTLs) in CD4 T cells among 260 genes with expression associated with viral set point.
(0.03 MB XLS)

Table S12 Genes identified in the current study that have been previously reported in genome-wide studies in Dengue.
(0.03 MB XLS)

Text S1 Supplementary materials
(0.88 MB DOC)
