# Supplementary figures and images for: Genome-Wide mRNA Expression Correlates of Viral Control in CD4+ T-Cells from HIV-1-Infected Individuals
Source: PLoS Pathog. 2010 Feb 26;6(2):e1000781. doi: 10.1371/journal.ppat.1000781 (PMC2829051; doi:10.1371/journal.ppat.1000781)

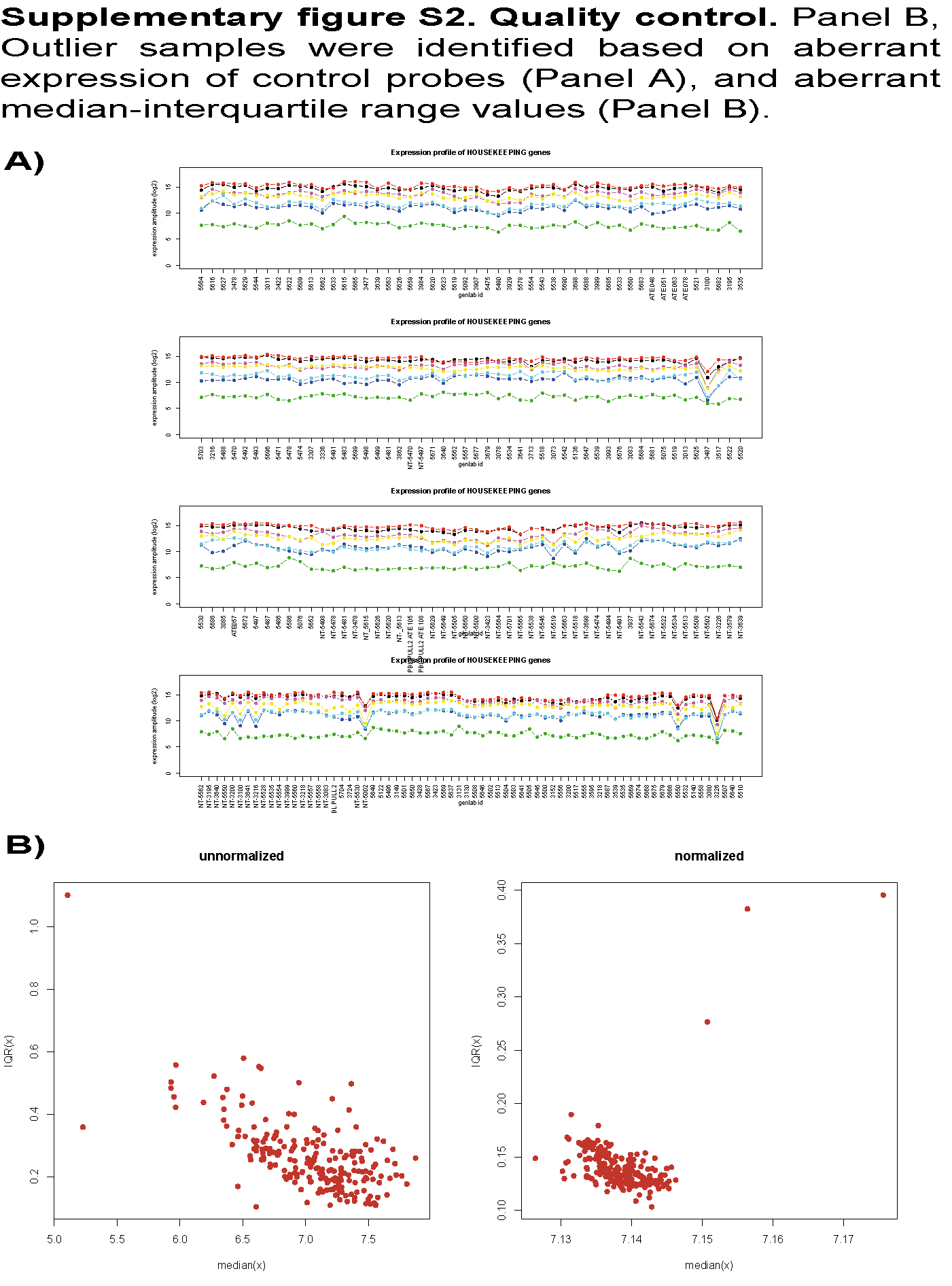

Supplement: Figure S2 — Quality control. Outlier samples were identified based on aberrant expression of control probes and aberrant median-interquartile range values. (0.34 MB TIF) [file ppat.1000781.s002.tif]
