# Supplementary material for: Genome-Wide mRNA Expression Correlates of Viral Control in CD4+ T-Cells from HIV-1-Infected Individuals
Source: PLoS Pathog. 2010 Feb 26;6(2):e1000781. doi: 10.1371/journal.ppat.1000781 (PMC2829051; doi:10.1371/journal.ppat.1000781)
Supplement: Text S1 — Supplementary materials (0.88 MB DOC) [file ppat.1000781.s015.doc]

**Supplementary materials**

**Genome-wide mRNA expression correlates of viral control in
CD4+ T-cells from HIV-1-Infected Individuals**

M. Rotger^1*^, K. Dang^2*^, J. Fellay^2*^, E.L. Heinzen^2^, S. Feng^2,3^, P. Descombes^4^, K.V. Shianna^2^,
D. Ge^2^, H. F. Günthard^5^, D.B. Goldstein^2§^, A. Telenti^1§^

The Swiss HIV Cohort Study and the Center for HIV/AIDS Vaccine immunology

^1^Institute of Microbiology, University Hospital and University of Lausanne, Switzerland; ^2^Institute for Genome Sciences & Policy, Duke University, USA; ^3^Department of Biostatistics and Bioinformatics, Duke University, USA; ^4^Genomics Platform, University of Geneva, Switzerland; ^5^Division of Infectious Diseases, University Hospital Zurich, University of Zurich, Switzerland

**Running head:** Genome-wide mRNA expression and HIV

* Equal contribution

Correspondence

David B Goldstein ([d.goldstein@duke.edu](mailto:d.goldstein@duke.edu)) or Amalio Telenti ([amalio.telenti@chuv.ch](mailto:amalio.telenti@chuv.ch))

**Content:**

- Demographic and experimental data on participants and samples.

- Definition of viral set point and of elite controllers

- Flow chart of samples

- Analysis of expression and splice variants in *OAS1*

**Demographic and experimental data on participants and samples.**

| Gender | Male | 139 (70.2) |
| --- | --- | --- |
| [N (%)] | Female | 59 (29.8) |
| Age  [mean (SD)] |  | 36.1 (10.6) |
| Ethnicity  [N (%)] | Caucasian | 198 (100) |
| Mode of HIV infection | Homosexual | 84 (42.4) |
| [N (%)] | Heterosexual | 60 (30.3) |
|  | Intravenous drug use | 49 (24.8) |
|  | Unknown | 5 (2.5) |
| Year of seroconversion*  [median (range)] |  | 2000 (1992-2005) |
| Number of days from seroconversion to sample date*  [median (IQR)] |  | 1156 (736-2126) |
| Number of days from sample date to transcriptome analysis (cryopreservation time)  [median (IQR)] |  | 616 (333-1448) |
| Number of viral load results used in setpoint calculation*  [median (IQR)] |  | 4 (2-8) |
| CD4 viability for samples included in final analyses  [median (IQR)] |  | 78.5 (70.5-85.3). |
|  |  |  |

*elite controllers without known date of seroconversion are excluded.

**Determination of Viral setpoint (Fellay et al. Science 317: 944-947, 2007)**

First step: Identification of all eligible patients that had 3 or more stable plasma HIV RNA results in the absence of antiretroviral treatment, and met one of the following criteria: [A] a valid seroconversion date estimation proven by documents or biological markers; or [B], for seroprevalent patients, VL data over a period of at least 3 years, diverging by no more than 0.5 log.

Second step: elimination of outlier VL on the basis of clinical or biological arguments

A. (only for the Euro-CHAVI cohort) Visual inspection of all the viral load data in relation to other clinical documentation to determine if any VL should be excluded because it was coincident with vaccination, immune-modulating treatment, major trauma or laboratory problems.

B. (for both cohorts) Elimination of VL data that was coincident with and subsequent to disease progression (defined as a CD4 count below 350 cells) or initiation of cART.

Third step: elimination of VL not reflecting the steady-state: 3 types of outliers were identified, corresponding to the 3-phasic evolution of HIV-1 viremia:

A. (for seroconverters only) VL measured before the set point has been reached, part of the initial peak of viremia observed during primary HIV infection: they have to be measured during the first year after seroconversion and have a value >0.25 log10 higher than average of subsequent VL.

B. VL measured during the late phase of the disease: for patients with a significantly ascending VL slope, we kept only the first 3 results for calculation of the set point.

C. VL measured during the set point period, but conflicting with other available results; possibly linked to unreported interfering conditions, laboratory errors, transcription or data-management errors: defined as VL >0.5log higher or lower than average of all remaining points.

Fourth step: calculation of the set point as the average of all remaining VL results.

**Definition of Elite controllers:**

• Asymptomatic HIV Infection over 10 year after seroconversion

• Longitudinal HIV RNA that includes a minimum of 3 determinations, in the absence of antiretroviral agents, which span at least a 12-month period.

• Plasma HIV RNA levels without ART that are below the level of detection for the respective assay (e.g., < 75 copies/ml by bDNA or < 50 by ultrasensitive PCR).

• Isolated episodes of viremia up to 1000 copies /ml as long as they are not consecutive and represent the minority of all available determinations.

**Flow chart of samples.** The exclusion of samples reflects the application of quality control criteria.

283 samples/198 participants

9 samples/3 healthy blood donors

247 samples/178 participants

9 samples/3 healthy blood donors

13 unviable samples

23 samples with degraded RNA

214 samples/164 participants

8 samples/3 healthy blood donors

34 samples failed gene expression analysis

4 samples were outliers

20 samples from unsuccessfully treated participants

190 samples/153 participants

8 samples/3 healthy blood donors

- Paired untreated/treated samples: 74/37 seroconverter participants
- Only untreated samples: 72/72 seroconverter participants
- Only treated samples: 28/28 seroconverter participants
- Elite controllers: 16/16 participants
- Samples from healthy blood donors: 8/3 healthy individuals

**Analysis of expression and splice variants in *OAS1***

The *cis*-acting screen identified rs3177979 in *OAS1* to be associated with expression. This SNP is in LD (r2=0.94) with rs10774671 that creates an alternative splice site leading to isoform 3 (Bonnevie-Nielsen et al. Am J Hum Genet 76: 623-633, 2005). Isoform 3, also known as p48 is associated with rs10774671. RT-PCR was performed in RNA samples from 10 members of the CEPH pedigrees representing the 3 genotypes (**Figure**). The presence of the G allele resulted in an amplicon of 577bp corresponding to the main isoform, while the presence of the A allele gave rise to 479bp (isoform p48) and 576bp fragments (isoform p52).

**Isoform amplification in 10 samples from CEPH pedigrees representing the 3 genotypes of rs10774671.**

**
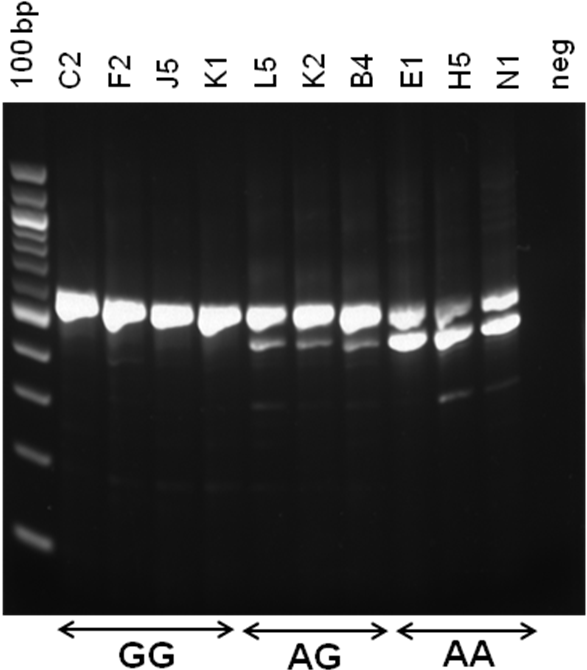
**
